# On Explaining Quantum Correlations: Causal vs. Non-Causal

**DOI:** 10.3390/e23050589

**Published:** 2021-05-10

**Authors:** Laura Felline

**Affiliations:** Independent Researcher, 00145 Rome, Italy; laura.felline@uniroma3.it

**Keywords:** quantum correlations, causal explanation, structural explanation

## Abstract

At the basis of the problem of explaining non-local quantum correlations lies the tension between two factors: on the one hand, the natural interpretation of correlations as the manifestation of a causal relation; on the other, the resistance on the part of the physics underlying said correlations to adjust to the most essential features of a pre-theoretic notion of causation. In this paper, I argue for the rejection of the first horn of the dilemma, i.e., the assumption that quantum correlations call for a causal explanation. The paper is divided into two parts. The first, destructive, part provides a critical overview of the enterprise of causally interpreting non-local quantum correlations, with the aim of warning against the temptation of an account of causation claiming to cover such correlations ‘for free’. The second, constructive, part introduces the so-called structural explanation (a variety of non-causal explanation that shows how the explanandum is the manifestation of a fundamental structure of the world) and argues that quantum correlations might be explained structurally in the context of an information-theoretic approach to QT.

## 1. Introduction

One of the main issues in the foundations of Quantum Theory (QT), and a motivation for the quest for a substitute to this theory, is the explanation of quantum correlations. At the basis of the problem lies the tension between the natural interpretation of correlations as the result of a causal process or the manifestation of a causal relation, and the fact that the underlying physics does not seem to adjust to the most essential features of a pre-theoretic notion of causation. In this paper, I argue for the rejection of the first horn of the dilemma, i.e., the assumption that quantum correlations are a causal phenomenon, calling for a causal explanation.

In the first part of the paper (Section 2), I provide a critical overview of the enterprise of causally interpreting quantum correlations. For simplicity, I will refer to EPR correlations, although the arguments illustrated throughout the paper (and unless differently specified) applies to the more general correlations of Bell’s theorem. (As an anonymous referee pointed out to me, the specific correlations considered in the EPR argument can indeed be provided a common cause explanation, see ([1], ch.3).) An important rationale for developing the non-causal view of quantum correlations is the acknowledgment of the history of unsuccessful attempts in the foundations of QT to reshape the notion of causality in such a way to cover quantum correlations. In a line: errare humanum est, perseverare diabolicum.

The efforts to adapt causation to the bizarre world of quantum mechanics led to countless variations and to the exploration of hypotheses that before QT were commonly considered at best exotic, at worst senseless. Just to make one notable example, quantum correlations have motivated the articulation of backward accounts of causation (e.g., [2]), where the causal influence travels back in time to determine the pair’s state at the source. To be sure, the unfamiliarity of such proposals is not reason to reject them; however, the piling critical literature about them nowadays witnesses their loss of attractiveness as a concrete solution to the problem. For instance, several arguments based on paradoxes have been put forward for the impossibility of backward causation (e.g., [3]); regardless of said arguments, reference [4] shows that such models fail to explain correlations both in indeterministic and in deterministic models. (See also [5], and [6] for a response.)

One could therefore insist that standard QT is a bad theory, and that the real causal explanation of quantum correlations can only be provided in a more satisfactory alternative theory. As we are going to argue, similar problems affect a causal interpretation of EPR correlations in the other main competing quantum theories: Bohmian mechanics and dynamical reduction theories (originally formulated by Ghirardi, Rimini, and Weber (GRW)).

Given the convolution of the literature, it cannot be a realistic aim of this paper to review all such existent or past causal interpretations of EPR, or to definitely disprove the overall project. Anyway, I could not think of a concrete way to argue for such a drastic conclusion. The aim of this section is to illustrate what is involved by the enterprise of causally explaining EPR correlations, and to warn against the mirage of an account of causation covering such correlations ‘for free’.

The second part of this paper (Section 3) introduces the so-called structural explanation, a variety of scientific explanation that occurs in fundamental physics and that consists of showing how the explanandum is the manifestation of a fundamental structure of the world. Here, I will argue that quantum correlations might be explained structurally and outline such a proposal by individuating in the structure of quantum information a fundamental structure that can explain correlations.

## 2. Non-Local Quantum Correlations and Causal Explanation

In the introduction to this paper, I have characterized the problem of explaining EPR correlations as pivoting on the tension between, on the one hand, a natural interpretation of correlations as the manifestation of a causal relation and, on the other, the fact that the physics underlying the correlations does not seem to adapt to the most basic features of physical causation.

In order to unpack this claim, we need to take a closer look to the problem of quantum non-locality. Since the formulation of Bell’s theorem, the latter has typically been illustrated as originating from the apparent contradiction between three probabilistic conditions: factorizability, lambda-independence, and empirical adequacy.

Starting from the latter of the three, empirical adequacy is simply the request that the theory have the same predictions as QT, which have been abundantly corroborated by experiments and are therefore considered to be here to stay. Lambda independence states that the choice of measurements in each round of the experiment is independent of the state of the particles at the emission, avoiding in such a way that quantum statistics are trivially recovered. This is also a very commonsensical assumption; therefore, its failure is complicated to defend. The backward causation accounts cited in the introduction are examples of this approach.

The last possibility is failure of the third condition. Factorizability expresses the claim that the positive or negative correlation between two independent (i.e., not connected by a cause–effect relationship) events is screened off by their common causes.

Let us take an EPR-Bohm experimental setting and denote with *λ* the complete state of the entangled system (particles 1 and 2) before measurement, with *a* and *b* the settings for measurement at the two sides of the experiment. Factorizability requires that, if λ is taken into account (which should screen off any correlation due to a common cause), the joint probability for the measurement results *l* and *r* of 1 and 2 factorizes in their individual probabilities, i.e., the joint probability for the two outcomes is equal to the product of the single outcomes’ probabilities.

*Factorizability* *P_a_*_, *b*_(*l, r*|λ) = *P_a_*^1^(*l|*λ) · *P_b_*^2^(*r|*λ)

At the basis of the condition of factorizability lies Reichenbach’s Common Cause Principle, according to which, once the correlation due to a common cause is taken into account, the remaining positive correlation originates from a direct causal influence between *l* and *r*. In an EPR scenario, a direct cause explanation implies superluminal causality, and this in turn suggests a problem with relativity. Failure of factorizability, therefore, implies that a common cause alone cannot account for the correlation between *l* and *r*, and an appeal to a superluminal causal influence is required instead. (The same conclusion is obtained in some counterfactual accounts of causation (e.g., [7]), since violation of factorizability also implies counterfactual dependence between distinct events).

A most prominent and time-honoured direction investigated in the literature with the intent to avoid a direct cause account, consists in weakening the requirement of a (common) common cause, to separate common causes for different counterfactual correlations between different measurement results [8]. However, it is a nowadays widely shared conclusion that appealing to a separate common cause does not solve the possible contradiction with Lambda-independence and empirical adequacy. (Keep in mind that such a conclusion exclusively concerns the context of non-relativistic QT, while the situation is different in algebraic quantum field theory (see for example [9])).

To sum up, the impasse just described originates in the contrast between two intuitive assumptions: first, that a correlation is always the manifestation of a causal relation (illustrated by Reichenbach’s principle); second, that in a relativistic world cause and effect cannot be space-like separated.

Notice that the dilemma persists only if one insists on relying to Reichenbach’s principle. According to more up to date accounts of causation by relevance, the causal interpretation of EPR correlations is less pressing than usually assumed. In particular, Hausman and Woodward [10] claim not only that the interventionist account—the currently dominant account of causation by relevance—does not cover the relationship between quantum correlations, but also that this should not be seen as a limitation, but rather as a merit of such an account of causation. Still, it might be argued that an account of causation by relevance (like the interventionist account) lacks the resources to solve the tension, in scenarios à la EPR, between the causal interpretation of correlations and the failure of essential features of causation. In other words, even if the contradiction between the three probabilistic principles above mentioned was considered solved, this is not sufficient to causally explain correlations. Rather, it is necessary to identify a causal element in the world and its representative in the theory, able to possibly relate the two measurement results. In this sense, the solution to the problem requires an explanation-how of the correlations, i.e., an explanation in terms of causation by production. (For an explication of the distinction between the concepts of causation by production and causation by relevance, see e.g., [11]).

The most accredited account of physical causation by production is the process account, according to which causal connections are instantiated by processes as continuous space-time lines [12]. For instance, in the Salmon–Dowe process theory of causation [13], a process is the world-line of an object, and causal processes are able to transmit a conserved quantity.

Due to its lack of spatio-temporal continuity, according to a process account of causation, standard QT is typically intended as providing a non-causal account of correlations ([12], pp. 247–259).

In Bohmian mechanics, alternative interpretations provide very different ontological characterizations to wave-function; however, the upshot is the same. On the one hand, neither the wave-function approaches (e.g., [14]) or the primitive ontology approaches (e.g., [15]) describe a continuous spatiotemporal process relating the two EPR measurement results, and therefore no action-at-a-distance. On the other hand, the two local dynamical processes described by the trajectories of the correlated particles alone do not account for their correlated measurement results (one needs to appeal to the role of the wave-function, as a law or as an entity in configuration space). A Salmon–Dowe’s causal explanation alone, therefore, cannot explain correlations.

When it comes to GRW, the spatiotemporal description of the collapse in this theory also changes dramatically depending on the ontological interpretation taken in consideration. Although also in this case, the upshot is the same as in Bohmian mechanics, i.e., incompatibility with the process account of causation, this case study requires a separate analysis in the mass density [16] and in the flash ontology [17].

In the first interpretation, the wave-function represents the density of mass in physical space and, at the instant of the collapse, matter instantaneously localizes in one point. Since the process of localization does not have a finite speed, it cannot be interpreted as describing matter ‘traveling’ in space-time, therefore the phenomenon is rather described as ‘delocalization’, a term that does not imply a continuous motion from point *a* to point *b*.

A simplified thought experiment originally formulated by Einstein (See [18] for a critical discussion of Einstein’s thought experiment) might be useful to illustrate this point. Let us put a quantum particle in a box with impermeable walls in such a way that the associated wave-function is confined to the box. Let us now split the box in two parts and transport one half of the box to Paris, the other to Tokyo. According to the density matter interpretation of GRW, as far as the particle is kept isolated inside the box, the field of density of matter is uniformly spread in the two halves of the box. When the half box in Paris is opened and, say, found empty, the matter located in Paris disappears and instantaneously reappears in Tokyo. Since traveling requires a certain speed, and this process takes a zero interval of time, the latter cannot be interpreted in terms of matter traveling from one point to another.

Returning to the EPR correlations, their explanation in the matter density interpretation of GRW also describes a process of localization of matter with no finite speed, which, being independent of the distance covered, cannot be described as matter ‘traveling’ in space, but only as matter disappearing in one place and reappearing in another. Being discontinuous in space-time, localization does not qualify as a causal process and, a fortiori, the GRW explanation of the correlations is not causal. The same problem replicates in the continuous spontaneous localization models [19] where, although collapse is not instantaneous, its duration is independent of the distance covered, and matter cannot therefore be conceived as ‘traveling’ from one place to the other, but only as ‘disappearing’ from one place and ‘re-appearing’ in another.

In the flash ontology interpretation, the only elements of the ontology are the so-called flashes, i.e., events of spontaneous localization whose probability of occurrence is represented by the wave-function. From this element alone, it follows that no continuous spatiotemporal process and, therefore, no causal process is to be found in this interpretation either.

Nowadays, the other main contestant as an account of causal explanation by production is mechanistic explanation ([20,21]), i.e., an explanation-how that implies the description of the processes underlying the phenomenon to be explained and of the entities that engage in such processes. Although mechanistic explanation is tightly entangled with the notion of causation, it is a matter of debate what kinds of mechanistic relations are causal (see e.g., [22]). To this respect, the only clear available account of causation in terms of mechanisms is Stuart Glennan’s account [23], according to which two events are related if they are mediated by a mechanism.

According to [24], at least three characteristic features of quantum systems in standard QT (indeterminateness of properties, indeterminateness of position, entanglement) are incompatible with mechanistic explanation.

First of all, while the behavior of a mechanism depends on the interaction of its parts in virtue of their dynamical properties, the vast majority of properties of a quantum object are virtually always indeterminate. Mechanistic explanations depending on such dynamical properties are therefore in general forbidden in quantum phenomena. Secondly, among such properties, one of the most important in mechanistic explanation, due to its role in the organization between a mechanism’s component parts, is the spatial location of such parts. In general, though, quantum objects are not describable as localized objects. As a consequence, and as we have already seen for the case of the process account of causation, this feature of mechanistic explanation is also generally problematic. Third, the behavior of a system composed of two entangled subsystems is mechanically unexplainable: in a mechanistic explanation, a complex system must be decomposable in the sum of its parts, but a mechanistic explanation requires the possibility of individuating parts with individual states.

Due to the spatiotemporal localization requirement, Glennan’s mechanistic account is also not available in Bohmian wave-function ontologies, given that the wave-function does not inhabit space-time and therefore cannot be part of a mechanism in Glennan’s sense. Additionally, the latter is also straightforwardly ruled out in primitive ontology interpretations of Bohmian mechanics, where the wave-function is a nomic entity that plays an explanatory role non-translatable in mechanistic terms.

Finally, in GRW, the very first requirement for a mechanism, i.e., the existence of a stable set of entities interacting with each other, is violated. In the flash ontology, the only elements of the ontology are events in space-time, and they clearly do not classify as mechanistic entities, let alone as entities interacting with each other. In the mass density ontology and in the continuous spontaneous localization models, there are no individual particles interacting with each other, but only one primitive stuff, a matter density field spread in physical space. In Glennan’s terms, the wave-function seems to behave rather as a fundamental entity (as it is, for instance, the electromagnetic field), whose behavior is not mechanistic. (Notice that a fundamental entity can be part of a mechanism when it interacts with other entities. This can very well be the case for the electromagnetic field, but not for GRW’s wave-function, which is the only stuff existing in the world).

## 3. Non-Causal Explanations

In the last decades, a flourishing literature has unveiled a variety of non-causal explanations in science and provided a philosophically rich soil to give flesh to the idea that quantum correlations are or could be explained non-causally.

A notable recent example is provided by Ismael’s and Shaffer’s [25] common ground explanation, a kind of metaphysical explanation covering several scientific fields, from logical explanations to mechanistic explanations in science. Currently, Ismael’s and Shaffer’s deserving proposal represents a viable metaphysical basis for a non-causal explanation of quantum correlations.

Others have tried to solve the problem with a less metaphysically-inclined approach, aimed rather at reflecting scientific practices and representations. This is the case of Structural Explanation, on which we are going to focus in the rest of this section.

The structural account of explanation was originally put forward by R.I.G. Hughes in [26]. Since then, various authors ([27,28,29]) have contributed to the characterization of this variety of explanation. In this section, we rely on the version put forward by Laura Felline [30,31,32].

This kind of explanation occurs in fundamental physics and exploits the formal features of the models displayed by the theories and works by showing how the formal representative of the explanandum counterfactually depends on other elements of the model.

Science displays countless explanations that work by exploiting a structure: from the topological explanations in the research on the stability of ecosystems [33] to the (also called) structural explanations in social sciences [34]. Not every such explanation, however, is structural in the sense adopted in this paper. In the above-mentioned topological explanations or in social sciences’ structural explanations, in fact, the explanandum instantiates a structure in the sense of an abstract, higher-level description that codifies the behavior of more fundamental entities. When a phenomenon is structurally explained, instead, it is shown to be a manifestation of a mechanistically fundamental physical structure, i.e., a structure that is not the higher-level description of more fundamental entities and processes [32].

This feature marks a difference with respect to other so-called mathematical explanations, in the sense that, if the explanatory structure is not mechanistically fundamental, there will always be the possibility of a more fundamental, causal, explanation of the same phenomenon.

A familiar example can help to concretely illustrate the relevance of such a difference in fundamental physics.

Before the formulation of Einstein’s relativity, length contraction and time dilation were thought to be the result of a causal process, whose explanation required the discovery of some new physical element, like an unknown force that ‘shrinks’ rods. The formulation of special relativity and the discovery that space-time has a Minkowskian—rather than Euclidean—geometry, provided a new, non-causal explanation of such phenomena, exploiting the geometric structure of relativistic space-time models; however, said non-causal explanation can be understood at least in two different ways.

In the first reading, the geometrical structure of space-time can and should be explained by more fundamental dynamical laws yet to be discovered. This is the so-called dynamical view of special relativity, according to which Minkowskian space-time can provide an explanation of phenomena in terms of second-order information about the world, yet, these explanations leave space for a more fundamental explanation in terms of ‘first-order’ causal information, like length contraction. Accordingly, such an explanation will be achieved by a more fundamental theory of matter that, once completed, will be able to ‘fully’ explain why rods contract, and why space-time displays such a geometry. Under this understanding, the geometrical explanations of special relativity are not structural explanations. Notice that in this case, the standard derivation of length contraction from the relativistic principles can count as an explanation according to the Deductive-Nomological model. However, under the assumption that the geometry of space-time (and length contraction with it) is the result of more fundamental dynamical laws, the explanatory significance of such inference changes, providing instead a further counterexample to the Deductive-Nomological model (cfr. [35]).

According to the main contestant to the dynamical view—that, following Harvey Brown and Pooley [35], we call the ‘orthodox view’—the structure of space-time is mechanically fundamental, i.e., it is not the abstract or higher-level description of the dynamical laws governing the behavior of matter at a more fundamental level. In this view, given the geometrical explanation showing how length contraction is the manifestation of a fundamental structure, there is no further, deeper causal/mechanistic/dynamical explanation to be found of this phenomenon. Such geometrical explanations are, in this case, structural explanations. In this case, the inference of length contraction from the relativistic principle also obtains a different status: rather than representing a mere logical deduction from a second-order law of nature, such an inference would show how phenomena are constrained by a fundamental principle.

Back to quantum correlations: assuming that quantum correlations are to be explained structurally rather than causally amounts to claim that, in QT, correlations are to be understood as the manifestation of a fundamental structure, rather than as the product of a dynamical process.

This idea is not new to the literature; indeed, it was suggested by Jeff Bub in the context of his early information-theoretic interpretation of QT (based on the Clifton, Bub, and Halvorson’s characterizing theorem [36]), where quantum information is understood as a new physical primitive:

“Just as Einstein’s analysis (based on the assumption that we live in a world in which natural processes are subject to certain constraints specified by the principles of special relativity) shows that we do not need the mechanical structures in Lorentz’s theory (the aether, and the behavior of electrons in the aether) to explain electromagnetic phenomena, so the CBH analysis (based on the assumption that we live in a world in which there are certain constraints on the acquisition, representation, and communication of information) shows that we do not need the mechanical structures in Bohm’s theory (the guiding field, the behavior of particles in the guiding field) to explain quantum phenomena.” ([37], p. 262)

Now, in order for the structure of information to provide a successful structural explanation of non-local quantum correlations, it is necessary to go beyond the minimal phenomenological interpretation of Shannon information, and assume that such a structure is fundamental in the sense that it is not explainable with the dynamical or constitutive details of underlying particles or waves. On the contrary, if we deny that the structure of information is a fundamental structure, then there must be a more fundamental story that infers/explains the structure of correlations, and we are back to square one: if there is such a story, then the problem of accounting for it with local dynamics appears again and we therefore still need to answer the question ‘how do quantum correlations come about?’.

In Bub’s early information-theoretic interpretation, not only is information a physical primitive, but its structure is the real and only object of QT. However, this last claim, i.e., that QT is only about information, has been disputed on various grounds. In particular, several arguments based on Wigner’s Friend scenarios (see for instance [38], but also [39]) have challenged the claim that black box approaches in information-theoretic interpretations provide a genuine solution to (or ‘explain away’) the measurement problem. (See [40] for a counterargument by Bub, and [41] for a reaction to Bub).

Notice that this does not necessarily rule out an information-theoretic structural explanation of correlations. On the contrary, one might maintain the existence of a mechanical story to be told about the determinateness of our results, and yet insist that correlations are structurally explained. The example of Special Relativity can once again come in handy to explain this point.

We have said that, in the orthodox interpretation, the explanation of length contraction appeals to the geometry of space-time and that, as a structural explanation, it is independent of the specific dynamical details of the concerned objects and their properties. Yet, if we have to explain the length contraction of a specific rod, within two specific inertial frames, we need to fill the explanation with initial conditions, e.g., the length (proper or not) of the rod. The explanation of the rod’s length cannot be provided within Special Relativity itself, but it must appeal to the dynamical history that produced an object of such length. This clearly does not undermine the geometrical explanation provided by Special Relativity, which, in the orthodox view, is not supposed to explain the proper length of the rod, but uniquely the different length it possesses in two different inertial frames. Moreover, the existence of a theory of matter that explains the length of the rod in no way undermines the fundamentality of the structure of space-time.

We suggest that a similar picture might be adequate in the case of information-theoretic approaches to QT and the explanation of correlations.

The structural explanation of non-local correlations in an information-theoretic approach involves the noncommutative algebraic structure of information and the reference to the fact that measurement results are determinate. (Let us take an EPR-Bohm experiment and the explanation of why, when Bob’s measurement got (say) spin-down, Alice’s measurement got spin-up. The explanation of a specific instance of non-local correlations (i.e., why Alice’s measurement result is spin-up when Bob registered spin-down?) in an information-theoretic approach involves the reference to the fact that Bob’s measurement yielded spin-down as an initial condition. In Bub’s view, the fact that the result is ‘down’ is explained away by the fact that the selection was random; however, that the result was determinate is not explained away and remains, therefore, a mere primitive fact [42]) Information-theoretic approaches, however, take such determinateness as a primitive, so they cannot explain it (Reference [42] may represent an exception to this rule). In order to achieve such an explanation, one might therefore appeal to a mechanical explanation involving the dynamical details that led to the determination of each result. As we have already seen in the case, e.g., of Bohmian mechanics, the dynamical story in itself does not explain the correlation; exactly as in the case of Special Relativity, therefore, the availability of a dynamical account of the determinateness of measurement results does not replace the need for a structural explanation of the correlations. Additionally, exactly as in the case of Special Relativity, the fact that one can tell a story about each individual result in the experiment, does not make the structure that constrains the correlations between such results less fundamental.

Different proposals have been put forward to go beyond the simplistic motto ‘QT is about information’ and that, in my view, leaves space for exploring the idea just hinted.

Koberinski and Müller [43] propose a ‘partial interpretation of QT’ as a principal theory of information, in the lines of [36], while they draw three possible completions to such a partial interpretation: ontic structural realism, subjective views of QT, and what we might call traditional ‘mechanical’ quantum theories (e.g., Everettian interpretations of QT, but also Bohmian mechanics). We here put aside metaphysical solutions à la ontic structural realism, whose evaluation requires considerations falling outside the scope of this paper. In participatory universe proposals, the structure of Hilbert spaces at the basis of the explanation would not count as a fundamental physical structure, but as a measure of belief, so that the explanation should be seen as belonging to the field of formal epistemology rather than physics (see [44]). Finally, the third option, that we call the ‘mechanical’ option, describes “a regime of our world that is currently empirically inaccessible, but which gives rise to the information-theoretic principles” ([43] p. 4). Depending of the specific relationship between the two (principle and constructive) interpretations, the mechanical theory can be seen as complementary to the information-theoretic reconstruction, or rather as ‘screening off’ the latter.

In his late work, ([1,45]) Bub has characterized his understanding of the claim ‘QT is about information’ as ‘QT is about probability’, still maintaining, though, the analogy with the explanatory Special Relativity:

“Heisenberg’s “re-interpretation” of classical quantities as noncommutative, or more specifically the entwinement of commuting and noncommuting observables, imposes objective pre-dynamic probabilistic constraints on correlations between events in a similar sense to how Minkowski space-time imposes kinematic constraints on events. The probabilistic constraints of the correlational structure provide the framework for the physics of a genuinely indeterministic universe. They characterize the structure of information for nonlocal correlations like Popescu-Rohrlich correlations, which can only occur between intrinsically random events.” ([1], p. 223)

In a recent account developing Bub’s interpretative line (so much that they baptized their approach ‘Bubism’), Janas, Cuffaro, and Janssen [42] advise that the divisive motto ‘QT is about quantum information’ is not interpreted as an ontological claim, but as a claim about “where the conceptual novelty of QT is located” (p. 139), that “this novel content can be located in the kinematical core of QT, in the structural constraints that QT places on our representations of the physical systems it describes” (ibid.). In this sense, the significance of QT as a theory of information is not to be found in its feature as a principal theory, as opposed to constructive theories, but rather in its kinematic content.

Janas, Cuffaro and Janssen adopt Pitowsky’s and Bub’s analysis of the measurement problem as including two distinct issues (the big and the small measurement problems), and Bub’s attempted solution to the big measurement problem provided in [40]; this, we think, opens their approach to the same potential objections to Bub’s approach and based on Wigners’ Friend scenarios [41]. However, they do acknowledge the existence of a measurement problem (the profound problem) that cannot be solved within the information-theoretic interpretation itself.

It should be said that neither Bub, Koberinski, and Müller, nor Janas, Cuffaro, and Janssen claim the need of a mechanistic solution to the measurement problem. In fact, in the view of such authors, the elimination of the measurement problem is a major motivation for the information-theoretic approach. However, their information-theoretic explanation of correlations does not rule out a mechanical account of measurement that ‘opens the black box’ and therefore leaves logical space for the latter.

It would be a matter of great interest to clarify whether and how such (structural?) information-theoretic explanations can cohabit with an ontological interpretation of quantum theory.

## 4. Conclusions

Two are the main points of this paper, that I wish I have successfully made. The first is a warning against the illusion of cheap solutions to the problem of causality in QT, especially in non-local correlations.

The second point I wanted to make is that there is a viable alternative for making sense of correlations, i.e., as a non-causal phenomenon, manifestation of a fundamental structure of the world.

In a nowadays-widely-cited passage, Michel Janssen provides a clear statement of the modern conception of the ‘orthodox’ view of kinematical phenomena, that includes length contraction, as well as inertia:

“It is a mistake to keep looking for further explanation of a phenomenon, once that phenomenon has convincingly been shown to be kinematical. What it means for a phenomenon to be kinematical, in the sense in which I want to use this term, is that it is nothing but a specific instance of some generic feature of the world, in the case of the phenomena examined in this paper instances of default spatio-temporal behavior. Unless one challenges the classification of the phenomenon as kinematical in this sense—and it is the universality of the relevant feature that will militate strongly against such reclassification—there is nothing more to learn from that particular phenomenon, neither about the specific system in which it occurs nor about the generic feature it instantiates.” [46].

In the same way, a full-fledged (information-theoretic, perhaps) structural explanation would show that the causal history of the entangled pair is superfluous in the explanation of EPR correlations.

After this paper was largely completed, I came across Reference [47], where Silberstein, Stuckey, and McDevitt provide a principal (structural?) account of EPR correlations “by applying a generalization of the relativity principle (“no preferred reference frame,” NPRF) to the measurement of Planck’s constant h to underwrite the qubit Hilbert space structure with its SU(2)/SO(3) transformation properties” (p. 21). Additionally, according to the authors, “the principle being posited herein does not require a solution to the measurement problem nor again does it necessarily beg for a constructive counterpart” (p. 11). I must reserve engagement with this important paper for future work.

## Data Availability

Data sharing not applicable.
